# 25,000 Years long seismic cycle in a slow deforming continental region of Mongolia

**DOI:** 10.1038/s41598-021-97167-w

**Published:** 2021-09-08

**Authors:** Laurent Bollinger, Yann Klinger, Steven L. Forman, Odonbaatar Chimed, Amgalan Bayasgalan, Ulziibat Munkhuu, Ganzorig Davaasuren, Tulga Dolgorsuren, Bayarsaikhan Enkhee, Demberel Sodnomsambuu

**Affiliations:** 1 CEA, DAM, DIF, Arpajon, France; 2 Institut de Physique du Globe de Paris, CNRS, Université de Paris, Paris, France; 3 Geoluminescence Dating Research Laboratory, Department of Geosciences, Baylor University, One Bear Place, Waco, TX 76798 USA; 4 Institute of Astronomy and Geophysics, Ulaanbaatar, Mongolia; 5 Mongolian University of Science and Technology, Ulaanbaatar, Mongolia

**Keywords:** Natural hazards, Tectonics, Seismology, Geomorphology

## Abstract

The spatial distribution of large earthquakes in slowly deforming continental regions (SDCR) is poorly documented and, thus, has often been deemed to be random. Unlike in high strain regions, where seismic activity concentrates along major active faults, earthquakes in SDCR may seem to occur more erratically in space and time. This questions classical fault behavior models, posing paramount issues for seismic hazard assessment. Here, we investigate the M7, 1967, Mogod earthquake in Mongolia, a region recognized as a SDCR. Despite the absence of visible cumulative deformation at the ground surface, we found evidence for at least 3 surface rupturing earthquakes during the last 50,000 years, associated with a slip-rate of 0.06 ± 0.01 mm/year. These results show that in SDCR, like in faster deforming regions, deformation localizes on specific structures. However, the excessive length of return time for large earthquakes along these structures makes it more difficult to recognize earthquake series, and could conversely lead to the misconception that in SDCR earthquakes would be randomly located. Thus, our result emphasizes the need for systematic appraisal of the potential seismogenic structures in SDCR in order to lower the uncertainties associated with the seismogenic sources in seismic hazard models.

## Introduction

The seismic behavior of faults in slowly deforming continental regions (SDCR) is a major source of uncertainty for seismic hazard assessment (e.g.^1^). Slow-slip faults show a variety of earthquake behaviors ranging from periodic seismicity to complex behavior alternating periods of clustered activity with seismic quiescence (e.g.^2,3^). In fact, it has been suggested that for some of the slowest slipping faults a single earthquake could occur with no distinguishable repeat pattern nor cumulative scarps^1^. These drastically different fault behaviors have been previously related to differences in loading rate, strength, healing rate, and interactions with external stressing rate, including the effects of lithospheric response to surface loads (e.g.^4–7^) and fault interactions within a spatial cluster of events. (e.g.^8,9^).

One major limitation in ascertaining which seismic behavior model might be most appropriate for SDCR is the limited number of paleoseismological sites recording a period long enough, from 10^4^ to 10^5^ years, to include a significant series of earthquakes. The earthquake record is often limited by the erosion rate that is too fast to allow preserving the geological records of these events (e.g.^10^). In most cases, only one or two events are preserved, making it difficult to examine the behavior of faults over multiple seismic cycles (e.g.^11^).

Here, we report a long paleoseismological archive in a SDCR by documenting a paleoseismic trench across the 1967 Mogod earthquake surface rupture that occurred in the low strain region of Mongolia. This surface rupture is exceptionally well preserved due to permafrost and low erosion rate.

## Background: seismotectonic setting

### Geodynamic context

**Figure 1:**
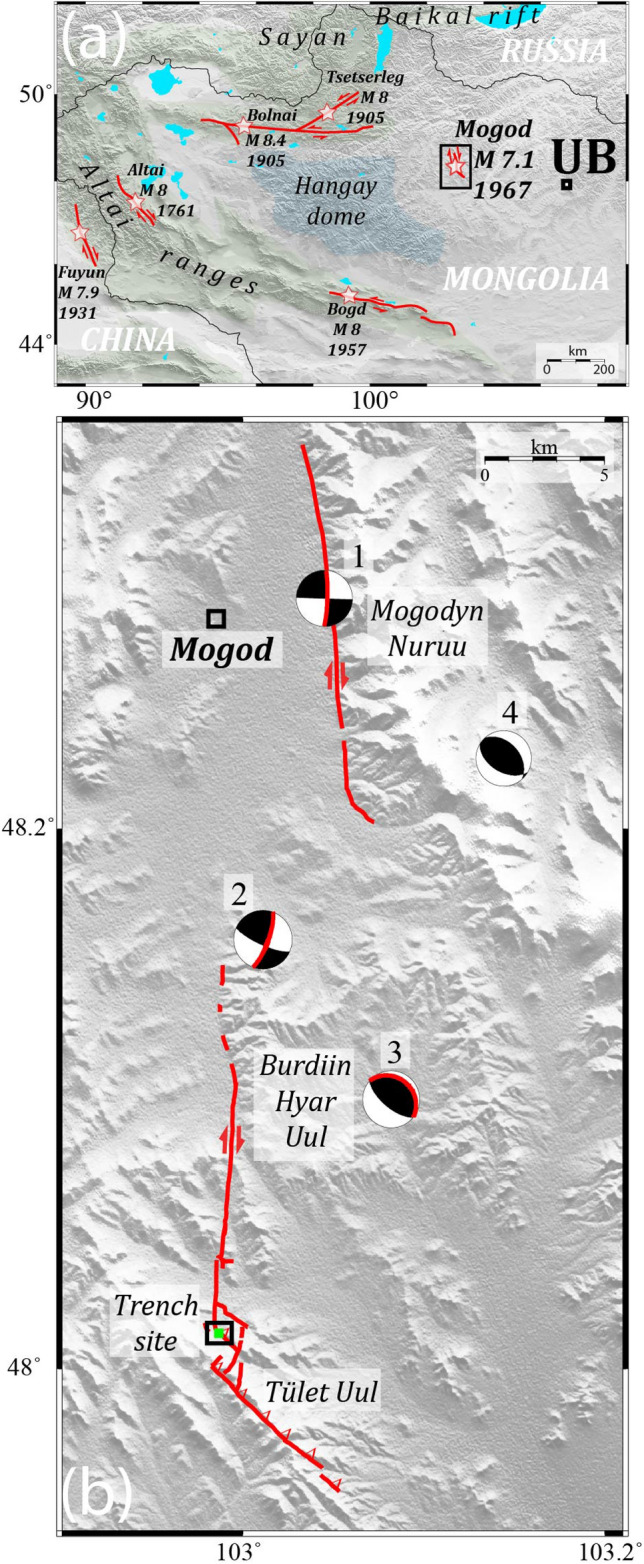
(**a**) Map of the large surface rupturing earthquakes of the last 300 years in Mongolia and vicinity. (**b**) Surface trace of the 1967CE rupture surveyed from high resolution images. Centroid Moment Tensors from Bayasgalan and Jackson, 1999. (1–2–3) label the 3 subsources of the Main shock in temporal order. (4) Is the largest aftershock that occurred on January the 20th 1967.

To the west of Hangay dome (a broad high-elevation low-relief topography in central-western Mongolia (Fig. 1a)), Mongolia is affected by large strike-slip faults that have been recognized as accommodating the northernmost part of the India-Eurasia collision^12^. Based on geologic and geodetic records, the slip rate on these different faults is estimated to be in the range of 1–3 mm/year (e.g.^13,14^). The stress build up related to this deformation is released by some moderate background seismicity, in addition to unusually large M8 strike-slip earthquakes, which seem to happen in temporal clusters triggered by postseismic viscoelastic stress transfer^8,9^. These large earthquakes have left spectacular surface ruptures scars along the principal fault systems including the Bolnai, Hovd, and Gobi-Altai systems^15–18^ (Fig. 1a). Farther North, the Baikal rift system accommodates localized extension at velocities locally larger than 3 mm/year (Refs.^19–21^). In contrast, the deformation appears more distributed in the Hangay dome^22^, a region where faults are sometimes only evidenced by their seismic activity^23,24^.

The areas of central and eastern Mongolia, east of the Hangay dome (Fig. 1a), are characterized by a low seismicity that often occurs during seismic swarms^25^. Fault slip rates, localization and recurrence time for large earthquakes remain mostly unknown in this part of Mongolia, usually described as a part of the Amurian plate, and recognized as a SDCR^26^. Indeed, the boundaries of the Amurian plate remain controversial due to the low seismicity rate, distributed faulting (e.g.^27–30^), and very low rates of deformation, ≤ 2.10^–8^ year^−1^ (Ref.^31,32^).

### The 1967 Mogod earthquake

The January 5th, 1967 Mogod earthquake occurred along the north-eastern edge of the Hangay dome (Fig. 1a). The seismic source of the Mw 7.1 earthquake was extensively studied using both seismological and field observations^33–35^. The earthquake surface ruptures are about 40 km-long in total, with numerous co-seismic displacements larger than 1 m^35–38^. The surface trace comprises three principal fault sections and dozens of secondary structures. Waveform inversions revealed that the earthquake could be decomposed in three seismic sub-events, which focal mechanism, magnitude, and location are consistent with the three surface rupture sections^34,35^. The northernmost fault section is mainly a North–South dextral strike-slip fault that ruptured along the western edge of the *Mogodyn Nuruu* mountain ridge (Fig. 1b). The second sub-event, also strike-slip, ruptured the central segment, generating mole tracks and tension cracks across the topography of a second ridge, the *Burdiin Hyar Uul* (Fig. 1b). In contrast, the third section corresponds to a series of thrust faults, which produced surface ruptures that follows the SE–NW trending ridge of *Tüleet Uul* (Fig. 1b).

**Figure 2:**
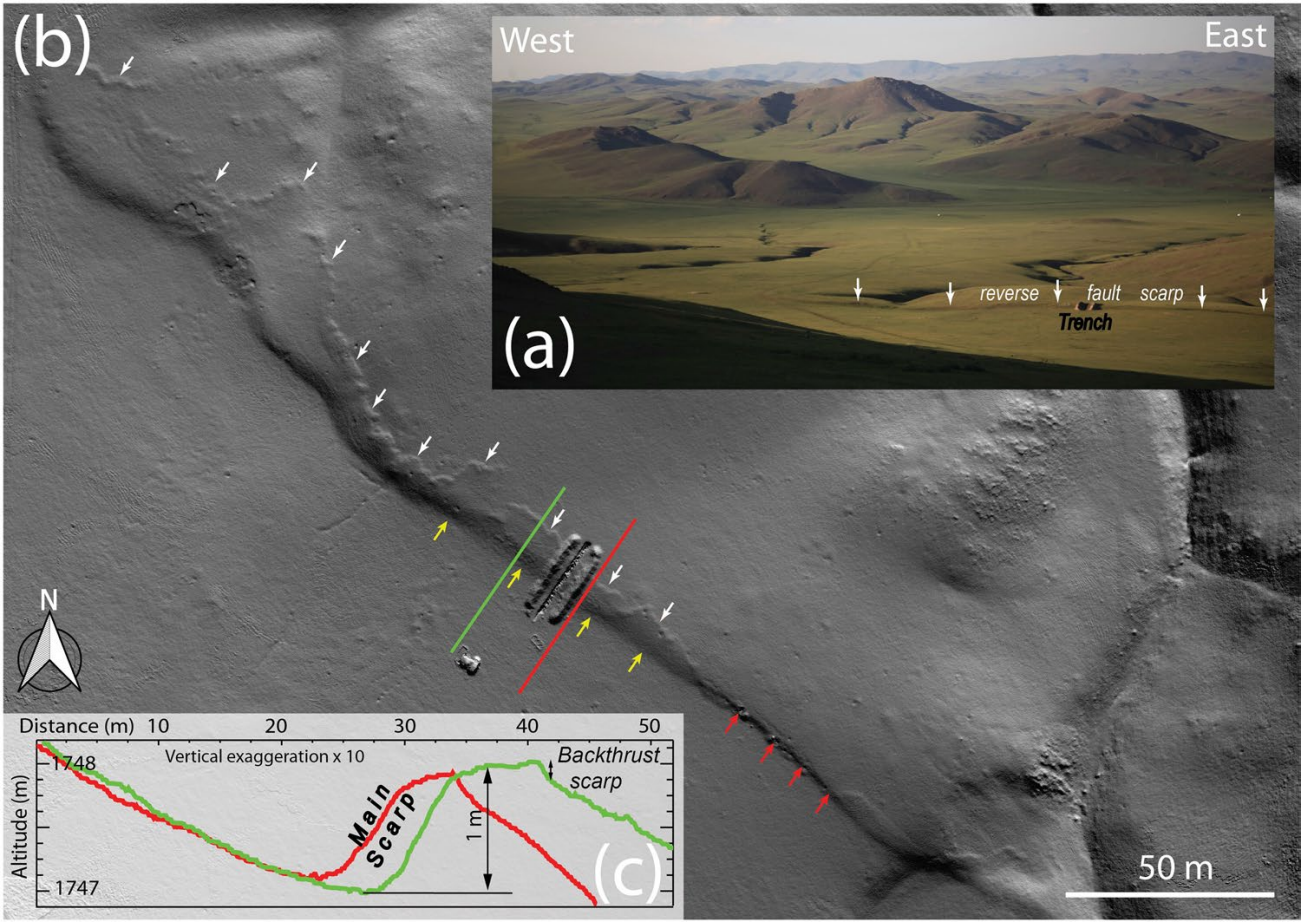
(**a**) Photograph of the trench and fault strand. (**b**) Shaded digital elevation model of the scarp at the trenching site—derived from UAV images. The yellow and red arrows point respectively toward the main flexural fault scarp and a section where the rupture on the main thrust reach the surface. The white arrows point toward the surface rupture of a secondary backthrust (**c**). Red and green topographic profiles through the Digital Elevation Model located on (**b**).

The geomorphic expression of the rupture is changing along strike, with the rupture trace probably being controlled by inherited structures at depth, enforcing segmentation of the fault system (e.g.^39^). The morphology of the ruptures at the junction between the central strike-slip section and the southern thrust section is complex, including two parallel reverse faults, which have clear surface rupture expression, in addition to many secondary ruptures. At the junction between the strike-slip and the thrust sections, the southernmost reverse scarp is located at the base of a large slope, facing up-slope and blocking sediments that go down the subdued drainage colleting surface wash (Fig. 2a,b). This southwest-facing scarp is ~ 1 m-high, with some lateral variations (Fig. 2b,c). The southeastern end of the scarp is well marked with the rupture reaching to the surface (Fig. 2b). Conversely, the morphology of the scarp is smoother further west, where the main fault is blind and the rupture is more characterized by a flexural scarp. However, parallel to the flexural scarp and north of it, a secondary 10 to 20 cm-high northeasterly facing scarplet is visible at the surface (Fig. 2b,c), which developed above a backthrust.

## Paleoseismological trench

**Figure 3:**
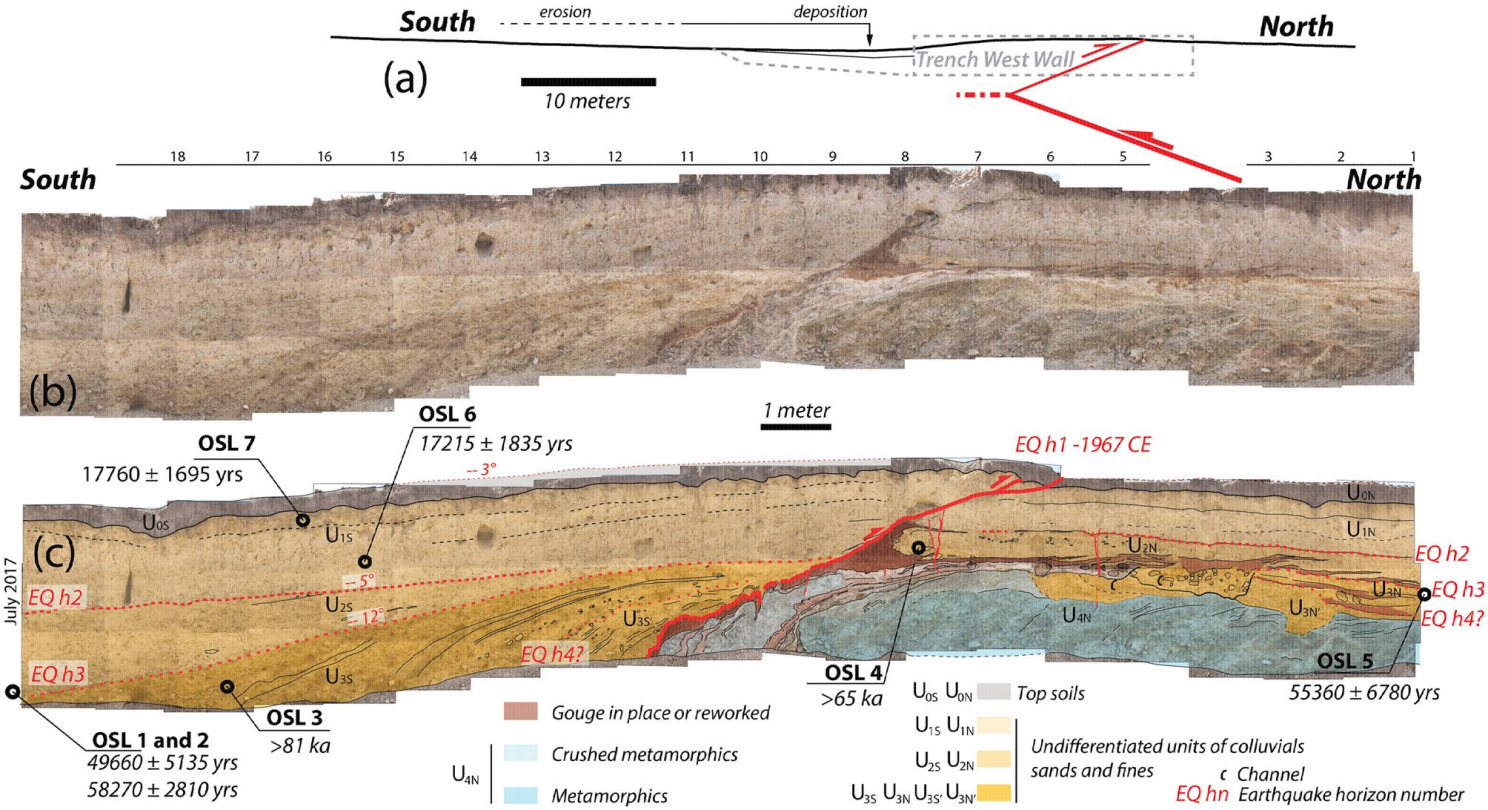
West Wall of the trench (**a**) Profile through the topography of Fig. 2 and emplacement of the trench wall. (**b**) Photomosaic and (**c**) Log of the west wall of the trench. July 2017. Minimum age Model OSL age from Table 1 are reported. Grey units U0 correspond to the topsoil offset by the 1967 surface rupture. It is deposited above U1 and U2 that correspond to undifferentiated units of sands and fines separated by an earthquake horizon (EQh2). This horizon corresponds to an erosional surface in the part located south of the backthrust and to a reworked red gouge in the northern part. EQh3 similarly separates U3 from U2. U3 sedimentary units were deposited above the metamorphic rocks of U4 (in blue).

We excavated a 20 m-long 2.5 m-deep trench at the *Tület Uul* site. The geological units exposed on the trench walls include fluvial and aeolian sediments, as well as metamorphic bedrock (Fig. 3). The fluvial sediments are fine, medium, and coarse sands, sometimes interbedded with matrix-supported gravels. The western wall was mapped in detail and subdivided into nine units based on lithology and geometry (Fig. 3). The eastern wall showed similar features confirming stratigraphic relations between the different units. However, due to limitation in time and poorer preservation we could not map it at the same level of detail. The main fault zone is not visible in the trench, but a backthrust branching off the main fault below the trench bottom reaches the ground surface. This backthrust, dipping 25° southward, is outlined by a reddish, gouge-rich, shear zone that forms a thrust sole that can exceed 20 cm in thickness (labelled “Gouge” on Fig. 3). All units visible in the trench are affected by this backthrust. Hence, they have been labeled U_xN_ and U_xS_ for units located respectively to the North and South of the backthrust.

The fault zone is characterized by a red-color shear zone that thickens downward, becoming more complex towards the base of the trench. Several folded layers and sheared chunks of bedrock have been incorporated into the fault zone, attesting of intense deformation.

Locally, the different units of the footwall are dragged and folded and include some gouge reworked by the fault, such as at the southern end of U_2N_, attesting of successive earthquakes (see next section).

The sedimentary units U_1_ to U_3_ can be found on both sides of the backthrust. South of the backthrust, in the hanging wall, the three units thicken gradually away from the scarp, following a growth strata geometry. Closer to the scarp, units onlap unconformably on top of each other along erosional contacts marked by coarser material (Fig. 3b). The successive dip angles change from 3° towards the south at the top of U_0S_, to 6° S between U_1S_ and U_2S_, and 13° S between U_2S_ and U_3S_, indicating incremental tilting.

North of the backthrust, in the footwall, the top layers are formed by fine sediments U_1N_ and U_2N_, almost identical to sediments across the fault. Below these units sits a fluvial unit, U_3N_, formed by coarse gravel to pebble in a sandy matrix that eroded in the shattered bedrock that forms the lower part of the wall. This unit contains 2 channels characterized by pebble and cobbles that eroded its older parts obliterating parts of the stratigraphic relations between the successive sedimentary units. The different sedimentary units (e.g. U_1N_ and U_2N_; U_2N_ and U_3N_) are consistently separated by a few cm-thick red layer that is interpreted to be some gouge-derived clay washed away from the fault zone along what was at that time the ground surface. This red clay is also found on top of the two channels, although stratigraphy has obviously been locally perturbed during emplacement of channels, and it is likely that the red clay that currently overlays the two channels has been remobilized one or several times, yielding complex depositional facies.

Eventually, the emplacement of each sedimentary unit U_1S_ to U_3S_ is interpreted to relate to rejuvenation of the flexural scarp during successive earthquakes that would dam the incoming sediments. Concomitantly, more gouge-derived clay would be washed away northward soon after the backthrust break to the surface. Thus, as detailed further down, our trench exposure suggests that there is evidence for at least three ground-breaking events at the *Tület Uul* trench site, including the 1967 Mogod earthquake.

## Age control on paleo-earthquake occurences

The earthquake horizon that corresponds to the most recent earthquake in 1967 is the current ground surface, where surface ruptures are still visible, suggesting that under the current climatic conditions both sedimentation and erosion rates are very low. Dating older units has proved difficult, as sediments are mostly devoid of any organic material usable for radiocarbon dating. Thus, 7 samples were collected in order to perform Optically Stimulated Luminescence (OSL) dating on sandy material.

Samples were collected by embedding 100 mm-long opaque metallic tubes into cleaned sections of trench western wall. In addition, bag samples were collected from around the OSL sample to determine the local dose rate, mineralogy, and particle size. The samples were then processed for OSL dating, following^40^ and analytical approaches as in Ref.^41^ (see also Supplementary Material). In most cases, dispersion of the equivalent dose *De* is 30% or larger. Such large dispersion might be due to different factors, including partial bleaching and mixing of the material. We therefore discuss ages considering a minimum age model (see sup. mat. for justification of such strategy). The seven dates distribute over a period from 17 to > 81 ka, spanning the last glacial period (Table 1, Fig. 3).

**Table 1:** Optically Stimulated Luminescence (OSL) ages on quartz grains sampled in the Mogod trenchsite.

Field number	Lab number	Aliquots	Grain size (μm)	Finite mixture D_e_ (Gy)	Minimum age model D_e_ (Gy)	Over-dispersion (%)	U (ppm)	Th (ppm)	K (%)	Cosmic dose rate (mGray/year)	Dose rate (mGray/year)	Finite mixture OSL age (year)	Minimum age Model OSL age (year)
MO17-OSL1	BG4574	5/50/60	250–150	146.12 ± 13.04	146.12 ± 13.04	37 ± 4	3.34 ± 0.01	6.98 ± 0.01	1.71 ± 0.01	0.22 ± 0.02	3.00 ± 0.06	48,650 ± 4310	49,660 ± 5135
MO17-OSL2	BG4579	11/36/40	250–150	156.90 ± 12.61	168.29 ± 6.41	31 ± 4	2.89 ± 0.01	6.98 ± 0.01	1.69 ± 0.01	0.22 ± 0.02	2.89 ± 0.15	54,370 ± 5195	58,270 ± 2810
MO17-OSL3	BG4576	20/23	150–100	> 250		NA	2.11 ± 0.01	9.11 ± 0.01	1.87 ± 0.01	0.22 ± 0.02	3.08 ± 0.15	> 81 ka	
MO17-OSL4	BG4578	36/40	250–150	> 195		31 ± 4	1.37 ± 0.01	5.29 ± 0.01	2.40 ± 0.01	0.22 ± 0.02	3.02 ± 0.15	> 65 ka	
MO17-OSL5	BG4575	6/31/31	250–150	149.22 ± 13.60	143.64 ± 17.64	31 ± 4	1.97 ± 0.01	6.18 ± 0.01	1.62 ± 0.01	0.25 ± 0.02	2.59 ± 0.14	57,500 ± 5370	55,360 ± 6780
MO17-OSL6	BG4573	6/47/51	250–150	39.50 ± 1.60	41.37 ± 4.29	61 ± 6	2.14 ± 0.01	5.04 ± 0.01	1.45 ± 0.01	0.25 ± 0.02	2.40 ± 0.06	16,440 ± 780	17,215 ± 1835
MO17-OSL7	BG4577	8/37/40	250–150	56.83 ± 2.21	50.45 ± 4.79	39 ± 5	2.41 ± 0.01	6.51 ± 0.01	1.73 ± 0.01	0.30 ± 0.03	2.84 ± 0.14	19,970 ± 885	17,760 ± 1695

See Supplementary Data material for methodology, full description and references.

At first order the stratigraphic order of ages fits the sample depth. OSL7 and OSL6 were sampled in unit U_1S_, the highest fine-sediments unit sampled for OSL, that yield consistent minimum ages of 17 ± 2 ka. Indeed, these ages overlap within one sigma errors and thus are statistically indistinguishable. The variation in mean ages reflects mostly systematic and random ages associated with OSL age calculation as outlined in the repository data material.

Downward, OSL1 and 2, sampled in well-defined clean sand pockets in U_2S_ give consistent ages of respectively 50 ± 5 and 58 ± 3 ka. These dates correspond probably to the activity of rill channels flowing along the scarp before the space in front of it get filled with new sediments. OSL3, collected below in unit U_3S_, yields an age > 81 ka. OSL5 was sampled north of the backthrust, in its footwall, at the top of a unit U_3N_ which stratigraphic relation to the top units is obscured by successive channeling and the deposition of two distinct levels of red clays derived from the gouge zone. This suggests that the top of U_3N_ was deposited slightly before the antepenultimate earthquake (earthquake horizon 3, Eqh3 in Fig. 3c). The age of top of U_3N_ is younger than 55 ± 7 ka (OSL5) and close to the age of OSL1 and OSL2, sampled at the base of U_2S_. Thus combining these observations we could bracket the age of EQ3 which event horizon is located between U_3_ and U_2_. Surprisingly, OSL4 that was sampled shallower and closer to the fault, in U_2N_, yields an age older (> 65 ka). Although we could not totally disregard this age (similarly to OSL3) this sample was far from ideal, as it was sampled in a rather heterogeneous drag fold hinge. Several factors might have biased age determination such sample saturation, or partial bleaching issues (see Supplementary Material for a complete report about OSL3 and OSL4 measurements). Hence, interpretation of this age should be handled cautiously.

## Discussion

**Figure 4:**
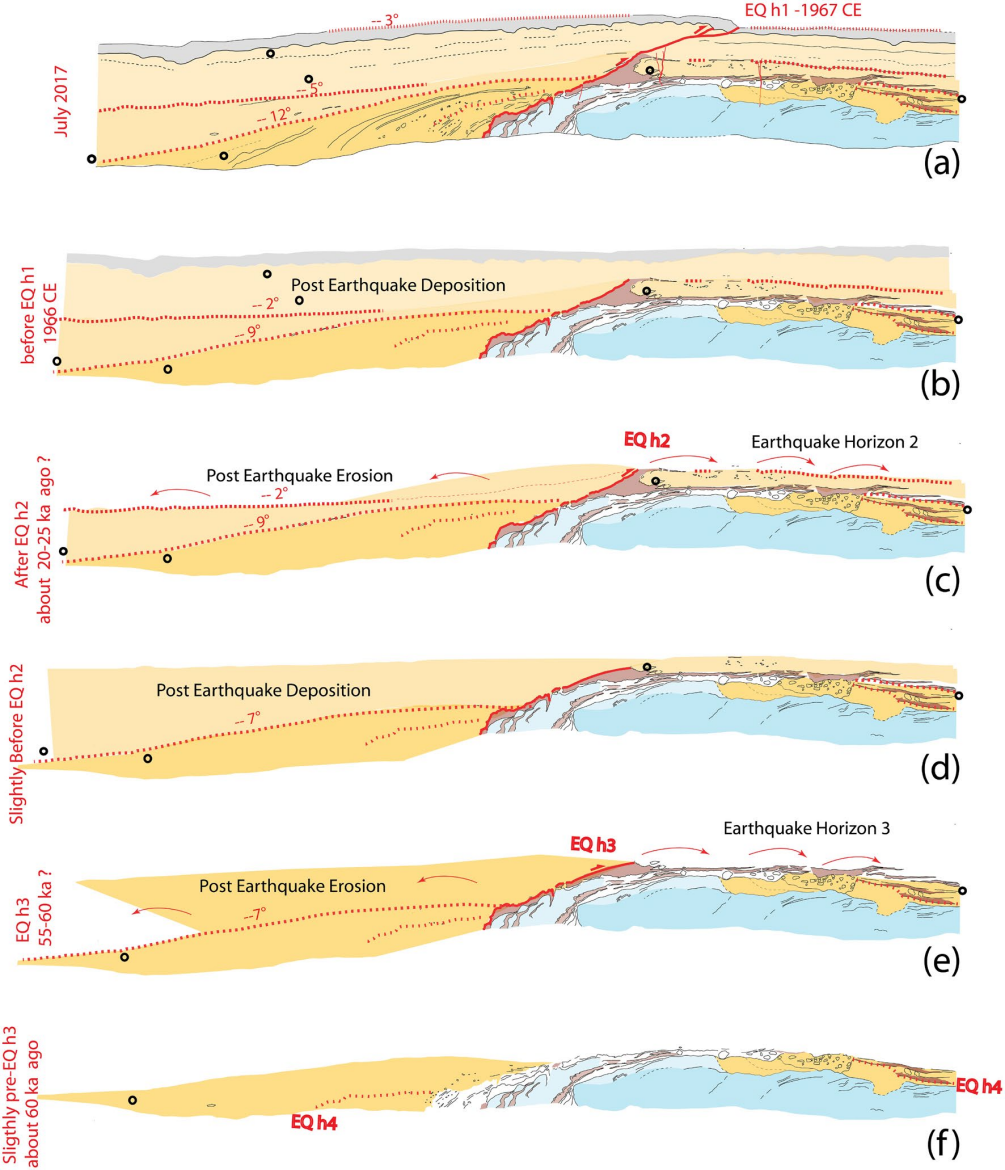
Schematic reconstruction. Incremental growth of the fold. Tilting of the frontal units.

Based on our trench exposure, we can propose a systematic earthquake-deformation scenario that repeated at least three times, including in 1967, and led to the current outcrop configuration (Fig. 4).

The tectonic expression of the thrust fault system varies along strike within a few meters, from a single sharp rupture along the main thrust fault plane, to blind thrusting and the activation of a backthrust pressure ridge, like what was already observed, for example, along the Spitak earthquake rupture^42^. During each earthquake, the blind thrust is activated that rejuvenates the main flexural scarp and uplifts the hanging wall by about 1 m (Figs. 2c, 4a,c,e). Similarly, the backthrust is also activated in the hanging wall, breaking to the surface. Up-slope fine-grained material, destabilized by earthquake ground shaking, quickly accumulates in front of the flexural scarp, which is facing mountain slope, transported either by wind or by surface wash. Some of the newly created scarp is also eroded, contributing as well to level off the trough in front of the scarp. At the same time, gouge-derived red clay is rinsed off from backthrust surface rupture and gets redeposited nearby on the ground surface (Fig. 4a,c,e).

Thus, the fault-related topography ends up being partly eroded and eventually buried by sediments coming down the slope of the mountain range (Fig. 4b,d). During the next earthquake, existing sediments get tilted and new sediments are deposited onlapping unconformably on top of older ones. The contact between these two sediment bodies combined with the thin clay layer, north of the backthrust, defines an earthquake horizon (Fig. 4c,e).

The last earthquake, in 1967, tilted the unit U_1S_ about 3° southward (Fig. 4a). Based on OSL6 and OSL7 ages about 17 ± 2 ka, and assuming that post-earthquake sediments get emplaced soon after the earthquake, in a couple of thousands of years, when destabilized sediments are most available, we propose that the penultimate event, Eq2, happened about 20–25 ka ago (Fig. 4c). The contact between U_1S_ et U_2S_ is tilted about 5° southward (Figs. 3c, 4a), roughly twice the tilt associated to the 1967 event, suggesting that locally the deformation was about the same amplitude. Based on samples OSL1, OSL2, and OSL5, and following the same reasoning as before, we propose that Eq3 happened about 55 to 60 ka ago. Indeed, such scenario implies that for some reason, not yet well understood, OSL4 gives an age older than the true deposition age. The existence of distinct steeply dipping matrix supported gravel beds at the base of U_3S_, next to the fault plane, and of a similar looking unit making an erosional contact on top of U_3S’_, north of the fault plane, suggest that a fourth event might be recorded in this trench. Hence, based on OSL3 this event would have happened more than 81 ka ago. Evidence for this fourth event, however, remains faint due to the poor signature of the tilt and erosional contact in the southern block. The multiple gouge-derived red clay horizons found between the base of U_2N_ and U_4N_ are short compare to the upper ones (Fig. 3c) and in fact they may reflect lateral transport of the clays associated to a single ante-penultimate event within successive channels rather than multiple events.

Age constrains in this trench only allow for a first order earthquake timing. However, assuming a systematic quick post-earthquake sedimentation across the scarp, and based on the ages available, our observation suggests that 3 to 4 Mogod-style earthquakes occurred on that fault, each time separated by 20 ka to 30 ka.

The exact amount of slip on the main blind thrust during the 1967 event remains unknown. However, considering the vertical deformation of the flexural scarp, ~ 1 m, and that about 10 cm to 20 cm of slip was accommodated by the backthrust, the total slip on the fault at depth cannot exceed about 1.5 m, independently of the fault dip at depth. Indeed, this is consistent with what was documented in the field^34^.

Thus, combining a first order return time for Mogod-style event of 25 ± 5 ka with a maximum slip of 1.5 m per event leads to a maximum slip rate on the Mogod fault of about 0.06 ± 0.01 mm/year estimated over several tens of thousands of years. This rate, which represents an upper bound, remains under the current detection threshold of geodesy.

### Implications for earthquake processes and hazard assessment in SDCR

The *Tület Uul* trench shows evidence for three to four earthquakes on the same structure, despite the absence of visible cumulative deformation in the surficial geomorphology. The climatic conditions allowed preserving a complete sedimentary record at our site, which is generally not the case in most of the slow-moving faults settings. The return time for Mogod-style earthquakes is on the order of a few tens of thousands of years. Thus, significant variations in climatic conditions have occurred between successive earthquakes, including the end of the last glacial period between the 1967 earthquake and the penultimate earthquake. Climatic conditions during the time period covered by our paleoseismological record include several episodes colder and wetter than today, during the last glacial period (e.g.^43^). Hence, in addition to the typical diffusion process that dominates erosion of scarps everywhere throughout Asia^44^, local erosional processes have likely been temporary emphasized by climatic changes. Eventually, given the very long time interval between successive earthquakes along the Mogod fault, and the small size of the co-seismic fault escarpment, these processes resulted in eroding evidence of previous surface ruptures and prevented the building of obvious significant cumulative scarps.

This is not specific to the Mogod earthquake area, and similar difficulties to find well-preserved Quaternary cumulative deformation have been reported elsewhere in central and northern Mongolia (e.g.^15,17,45^). This trench, however, demonstrates that even in SDCR with low rates of deformation, the deformation could localize on specific structures where deformation is accommodated through successive earthquake cycles despite the absence of visible cumulative scarps. In fact, absence of cumulative topography makes it difficult to assess the actual length of the fault structure beyond the section that did rupture most recently. Hence, it suggests that in SDCR a special attention should be paid in localizing such fault zones and trying to describe longer paleoearthquake time series (e.g. Refs.^45–48^), as they bear special significance in term of assessing seismic hazard.

In the case of Mongolia, the Mogod earthquake came in 1967, at the end of an earthquake sequence that ruptured 4 major faults during 4 magnitude M8 earthquakes, between 1905 and 1957^15,17,18,48–51^. All together, these 4 events released in about 50 years the stress equivalent to a loading rate of 50 mm/year (Ref.^52^), about 6 times what is actually measured by geodesy across Mongolia^13^. Thus, it has been proposed that this unusual sequence resulted from specific fault interactions and visco-elastic relaxation effects of one event that would lead to trigger the next one^8,9^, ending up in a major spatio-temporal cluster of events. The question of existence of similar earthquake clusters earlier in time remains unsolved yet. At best, it has been shown that return time for M8 earthquakes along the Bulnai fault is on the order of 3 ka to 4 ka^15^. Similar time scale has been suggested for the Fuyun fault^48^ although timing is poorly constrained. Our trench shows that Mogod-style earthquakes follow a different pattern with a repeat time of the order of several tens of thousands of years. The XXth century earthquake cluster in western central Mongolia, however, has certainly contributed to the loading the Mogod fault and might have hastened the Mogod earthquake. Hence, it emphasizes that for active faults with low loading rate, the contribution to fault loading of far seismic sources could be more significant than in faster deforming regions, where long-term tectonic loading dominates, and should therefore be considered thoroughly in SDCR seismic hazard assessment.

## Supplementary Information

Supplementary Information.
