# Supplementary material for: 25,000 Years long seismic cycle in a slow deforming continental region of Mongolia
Source: Sci Rep. 2021 Sep 8;11:17855. doi: 10.1038/s41598-021-97167-w (PMC8426405; doi:10.1038/s41598-021-97167-w)
Supplement: Supplementary file 1 — Supplementary Information. [file 41598_2021_97167_MOESM1_ESM.docx]

*Supplementary data material for*

**25,000 Years Long Seismic Cycle in a Slow Deforming Continental Region of Mongolia**

Laurent Bollinger^1,*^, Yann Klinger^2^, Steven L. Forman^3^, Odonbaatar Chimed ^4^, Amgalan Bayasgalan^5^, Ulziibat Munkhuu^4^, Ganzorig Davaasuren^4^, Tulga Dolgorsuren^4^, Bayarsaikhan Enkhee^4^, Demberel Sodnomsambuu^4^

^1^-CEA, DAM, DIF, Arpajon, France

^2^-Université de Paris, Institut de physique du globe de Paris, CNRS, Paris, France.

^3^-Geoluminescence Dating Research Laboratory, Department of Geosciences, Baylor University, One Bear Place, Waco, TX 76798, USA

^4^-Institute of Astronomy and Geophysics, Ulaanbaatar, Mongolia

^5^-Mongolian University of Science and Technology, Ulaanbaatar, Mongolia

*Email : Laurent.bollinger@cea.fr

**Contents of this file**

Text on the Optical-stimulated Luminescence (OSL) dating of quartz grains from fault-related sediments.

Supplementary Table S1

Supplementary Figure S1

Single aliquot regeneration (SAR) protocols (ref. 1, 2) were used in this study to estimate the apparent equivalent dose of the 150-250 or 150-100 μm quartz fraction for 60 to 23 separate aliquots (Table S1). Each aliquot contained approximately 20 to 80 quartz grains corresponding to a 1-millimeter or less circular diameter of grains adhered (with silicon) to a 1 cm diameter circular aluminum disc. The sands analyzed were mineralogically immature with SiO2 content of 50% to 67% for the non-carbonate fraction and are predominantly moderately to poorly sorted silty-sand with grains of variety of feldspars (ca-rich), mafic and other lithic fragments and quartz. The quartz fraction for a specific grains-size range (e.g. 150-250 µm) with sieving was isolated by density separations using the heavy liquid Na–polytungstate, and at least one 40-minute immersion in hydrofluorosilicic acid (HF 40%), to etch the outer ~10 µm of grains, which is affected by alpha radiation (ref. 3). The isolated quartz fraction was rinsed finally in HCl (10%) to remove any insoluble fluorides.

The optical purity of quartz separates was tested by exposing aliquots to infrared excitation (1.08 watts from a laser diode at 845 ± 4 nm), which often preferentially excites feldspar minerals. If this test indicated feldspar contamination, the HF 40-minute immersion was repeated. Another safe-guard of quartz grain purity was examination of a small subsample with a visualized petrographic microscope and by Raman spectroscopy. If a subsample exhibited > 1% non-quartz minerals, particularly feldspar minerals, the sample was retreated with density separations, HF soaking for an additional 20 or 40 minutes, sieved again and recheck for grain purity. The resultant final, prepared samples have a quartz purity of > 99% and showed weak emissions (<400 counts/second) with infrared excitation at or close to background counts.

An Automated Risø TL/OSL–DA–15 system was used for SAR analyses. Blue light excitation (470 ± 20 nm) was from an array of 30 light-emitting diodes that deliver ~15 mW/cm2 to the sample position at 90% power. Optical stimulation for all samples was completed at an elevated temperature (125° C) using a heating rate of 5 °C/s. All SAR emissions were integrated for the first 0.8 s of stimulation out of 40 s of measurement, with background emissions integrated for the last ten seconds of data collection, for the 30 to 40 s interval. The luminescence emission for all quartz fractions showed a dominance of a fast component with > 90% diminution of luminescence after 4 seconds of excitation with blue light (see Fig. S1). The fast ratio was calculated for natural emission and the equivalent emissions for a regenerative dose for each aliquot (ref. 5). Aliquots with a fast ratio of <15 were removed from the final equivalent dose analysis; also, aliquots with infrared depletion ratio of > 5%.

A series of experiments was performed to evaluate the effect of preheating at 160, 180, 200, 220, and 240 °C on isolating the most robust time-sensitive and thermal transfer emissions of the regenerative signal prior to the application of SAR dating protocols. These experiments entailed giving a known dose (40 Gy) and evaluating which preheat resulted in recovery of this dose. There was concordance with the known dose (40 Gy) for preheat temperatures above 180 °C with an initial preheat temperature used of 200 °C for 10 s in the SAR protocols. A second “cut heat” at 180 °C for 10 s was applied prior to the measurement of the test dose. A final heating at 260 °C for 40 s was applied to minimize carryover of luminescence to the succession of regenerative doses. A test for the reproducibility of the radiation-induced SAR ratio (Lx/Tx) was also performed by giving the same beta (90Sr/90Yt) source radiation exposure for the initial and the final regenerative dose and evaluating the concordance of the SAR ratios, which should be within 10% (ref. 1, 2).

The SAR protocols were used to resolve equivalent doses for seven samples (Table 1). The statistical significance of an equivalent dose population was determined for fifty to thirty-one quartz aliquots for five out of the seven samples, that were not at dose saturation (Table 1). Aliquots were removed from analysis if the fast ratio was < 20 (ref. 5), the recycling ratio was not between 0.90 and 1.10, the zero dose was > 5% of the natural signal or the error in equivalent dose determination was >10% (ref. 1, 2). The regenerative growth curves are modeled by using the exponential plus linear form. Error analysis for equivalent dose calculations for individual quartz aliquots assumed a measurement error of 1% with 2000 Monte Carlo simulation repeats to better characterized errors. Recuperation is < 3% for all samples, which indicates insignificant charge transfer during the measurements. These favorable luminescence characteristics for most aliquots indicate that credible equivalent dose values for these sediments can be determined by the SAR protocols.

The equivalent dose (De) distributions were log normal and exhibited overdispersion values between 31 and 61%. An overdispersion percentage of a De distribution is an estimate of the relative standard deviation from a central De value in context of a statistical estimate of errors (ref. 6, 7). A zero overdispersion percentage indicates high internal consistency in De values with 95% of the De values within 2σ errors. Overdispersion values < 20% are routinely assessed for quartz grains that are well solar reset, like aeolian sands (e.g., ref. 8) and this value is considered a threshold metric for calculation of a De value using the central age model of ref. 7. Overdispersion values >20% indicate mixing or grains of various ages or partial solar resetting of grains; the minimum age model (MAM; four parameters) may be an appropriate statistical treatment for such data. The MAM can effectively model the De components that are time dependent and inherited (ref. 4, 9). However, some studies have concluded that overdispersion values between 20 and 32% may reflect a signal De population, particularly if the De distribution is symmetrical, with the dispersion related to variability associated with micro-dosimetry and/or sedimentary processes (e.g., ref. 10). We consider overdispersion values >20% to indicate non-age-related processes such as post-depositional mixing of grains of various ages, partial solar resetting of grains or complex microdosimetry. Another complimentary statistical approach for the analysis of De populations with high overdispersion values is the Finite Mixture Model (FMM, ref. 6). This numeric analysis is an appropriate statistical treatment to identify De populations associated with single grain or very small aliquots, as used in this study (ref. 6). We present ages by both numeric treatments as a test of De robustness.

The environmental dose rate (Dr) is critical measurement for calculating a luminescence age. The Dr is an estimate of the exposure of quartz grains to ionizing radiation from the decay of the U and Th series, 40K, Rb and cosmic sources during the burial period. The U, Th, Rb and K concentrations are determined on the bulk sediment by inductively coupled plasma mass spectrometry by ALS Laboratories, Reno, NV. The beta and gamma doses were adjusted according to grain diameter to compensate for mass attenuation for the dose rate (ref. 11). Beta and gamma attenuation coefficients for 250 to 150 µm are 0.876 and 0.999, respectively (ref. 12). A cosmic ray component, considering location, elevation and depth of strata sampled was calculated to be between 0.16 and 0.20 mGy/yr, which includes the soft component (ref. 9, 13). There is uncertainty in assessing the moisture content of a sample during the burial period. We estimated moisture content (by weight) from present values, particle size characteristics and in reference to field indicators on the height of the water table. The datum year for all OSL ages is AD 2010.

A total of seven quartz extracts from corresponding sediment samples were dated by the SAR protocols (ref. 2) as outlined in ref. 14 for the Hunshandake Sandy Lands, Inner Mongolia northern China. Two of the seven extracts showed a natural OSL signal that was at dose saturation (~ 200-250 Gy) and yielded non-finite, possibly minimum limiting OSL ages (Table 1 and Fig. S1). The remainder of the extracts gave finite ages, though with elevated overdispersion values between 31 and 61%. These high values indicate that there are multiple De grain populations and the Minimum Age Model and Finite Mixture Model are the appropriate statistical analyses to resolve a finite age estimate (ref. 4, 6). OSL ages by these two different statistical analyses yield similar ages that overlap at 95% confidence level (Table S1).

General comment:

All OSL and other geochronological information when compared should be considered at one or two sigma error limits. OSL7 and OSL6 ages overlap at one sigma errors (68% of the variability) and thus are statistically indistinguishable. The variation in mean ages reflects mostly systematic and random ages associated with OSL age calculation as outlined in ref. 9.

Specific comment on OSL3 and 4:

OSL 4 (and 3) yielded an infinite age estimate partially related to the high dose rate of > 3 mGray/yr and dose saturation response in the regenerative growth curve, with SAR analysis for the infinite equivalent dose (>195 Gy). This infinite (greater than) age determination may reflect an older (>65 ka) age for these sediments and/or reworking of partially or un-solar reset grains into the sampled sediments, possibly with formation of fault gouge.

| **Field number** | **Lab number** | **Aliquots** | **Grain size (μm)** | **Finite Mixture**  **De (Gy)** | **Minimum Age Model**  **De (Gy)** | **Over- dispersion (%)** | **U (ppm)** | **Th (ppm)** | **K (%)** | **Cosmic**  **Dose rate**  **(mGray/yr)** | **Dose rate (mGray/yr)** | **Finite Mixture OSL age (yr)** | **Minimum age Model OSL age (yr)** |
| --- | --- | --- | --- | --- | --- | --- | --- | --- | --- | --- | --- | --- | --- |
| MO17-OSL1 | BG4574 | 5/50/60 | 250-150 | 146.12 ± 13.04 | 146.12 ± 13.04 | 37 ± 4 | 3.34 ± 0.01 | 6.98 ± 0.01 | 1.71 ± 0.01 | 0.22 ± 0.02 | 3.00 ± 0.06 | 48,650 ± 4310 | 49,660 ± 5135 |
| MO17-OSL2 | BG4579 | 11/36/40 | 250-150 | 156.90 ± 12.61 | 168.29 ± 6.41 | 31 ± 4 | 2.89 ± 0.01 | 6.98 ± 0.01 | 1.69 ± 0.01 | 0.22 ± 0.02 | 2.89 ± 0.15 | 54,370 ± 5195 | 58,270 ± 2810 |
| MO17-OSL3 | BG4576 | 20/23 | 150-100 | >250 |  | NA | 2.11 ± 0.01 | 9.11 ± 0.01 | 1.87 ± 0.01 | 0.22 ± 0.02 | 3.08 ± 0.15 | >81 ka |  |
| MO17-OSL4 | BG4578 | 36/40 | 250-150 | >195 |  | 31 ± 4 | 1.37± 0.01 | 5.29 ± 0.01 | 2.40 ± 0.01 | 0.22 ± 0.02 | 3.02 ± 0.15 | >65 ka |  |
| MO17-OSL5 | BG4575 | 6/31/31 | 250-150 | 149.22 ± 13.60 | 143.64 ± 17.64 | 31 ± 4 | 1.97± 0.01 | 6.18 ± 0.01 | 1.62 ± 0.01 | 0.25 ± 0.02 | 2.59 ± 0.14 | 57,500 ± 5370 | 55,360 ± 6780 |
| MO17-OSL6 | BG4573 | 6/47/51 | 250-150 | 39.50 ± 1.60 | 41.37 ± 4.29 | 61 ± 6 | 2.14 ± 0.01 | 5.04 ± 0.01 | 1.45 ± 0.01 | 0.25 ± 0.02 | 2.40 ± 0.06 | 16,440 ± 780 | 17,215 ± 1835 |
| MO17-OSL7 | BG4577 | 8/37/40 | 250-150 | 56.83 ± 2.21 | 50.45 ± 4.79 | 39 ± 5 | 2.41 ± 0.01 | 6.51 ± 0.01 | 1.73 ± 0.01 | 0.30 ± 0.03 | 2.84 ± 0.14 | 19,970 ± 885 | 17,760 ± 1695 |

Table S1: Optically Stimulated Luminescence (OSL) ages on quartz grains sampled in the Mogod trenchsite. Field and lab number identify the samples. Aliquots column reports aliquots measured, used and that define lowest most De population by Finite Mixture Model (ref. 6). Finite Mixture and Minimum Age models De correspond to equivalent dose (De) calculated on a pure quartz fraction with ultra-small aliquots with 20-80 grains/aliquot and analyzed under blue-light excitation (470 ± 20 nm) by single aliquot regeneration protocols (ref. 1). Equivalent dose (De) was calculated by the Finite Mixture Model (ref. 6) and the four parameter Minimum Age Model (ref. 4). Overdispersion values reflects precision beyond instrumental errors; values of ≤ 25% (at 1 sigma limit) indicate low dispersion in equivalent dose values and a unimodal distribution. Values > 25% are associated with mixed equivalent dose signature reflecting multiple grain populations or partial solar resetting. U, Th, Rb and K content analyzed by inductively-coupled plasma-mass spectrometry analyzed by ALS Laboratories, Reno, NV; and includes dose contribution from Rb and a moisture content 5 ± 2%. Dose rate includes also a cosmic rate calculated from parameters in ref. 13 and includes soft components. The systematic and random errors associated with the OSL age include systematic and random errors calculated in a quadrature at one standard deviation by the Luminescence Dating and Age Calculator at <https://www.baylor.edu/geosciences/index.php?id=962356> . Datum year is AD 2010.


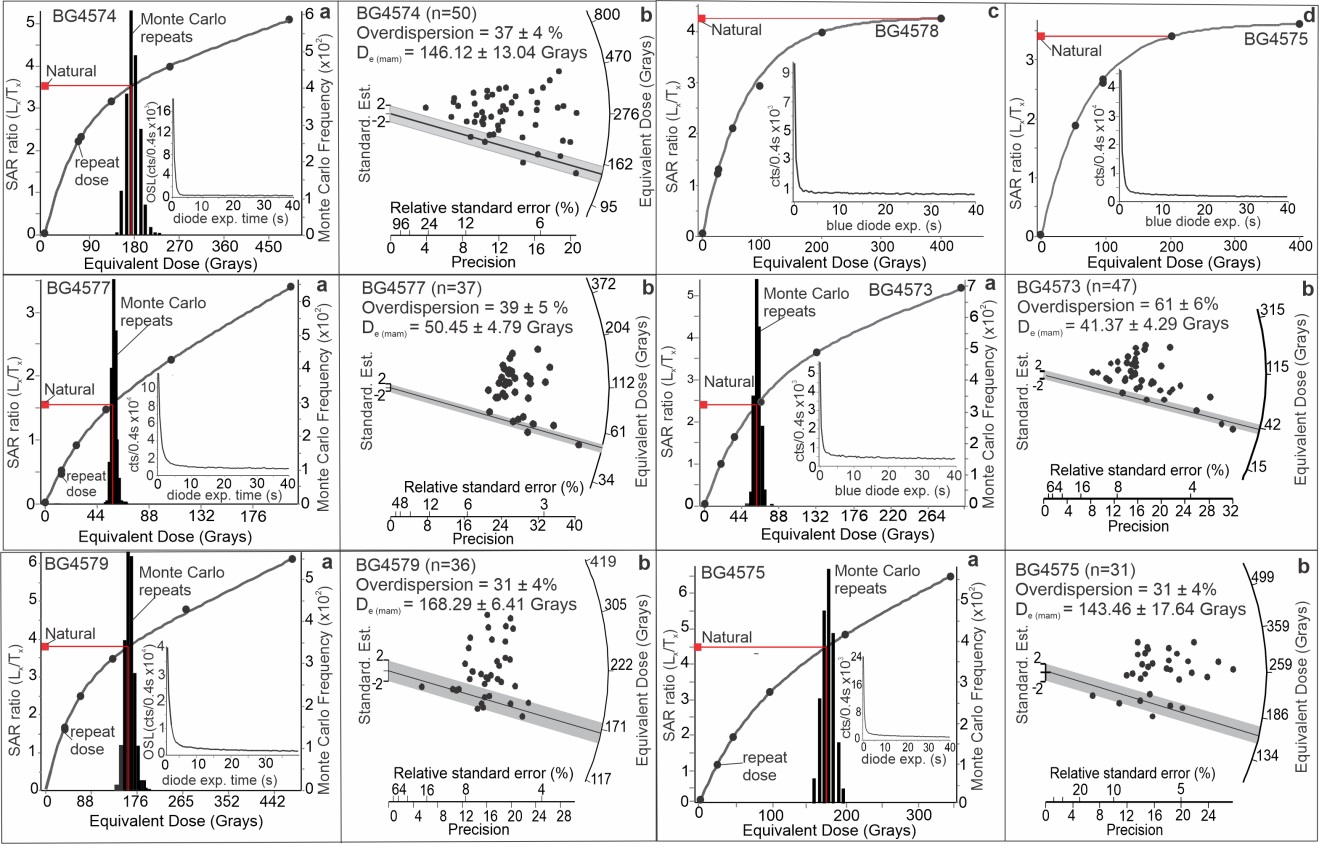


Fig. S1. (a) OSL regenerative growth curves for quartz grains for samples BG4573, BG4574, BG4575, BG4577, and BG4579 with inset figure showing representative shine-down curve for a natural emission. (b) Radial plots of Equivalent Dose (De) values for quartz ultra-small aliquots. Shown is the two sigma De range for the Minimal Age Model (MAM) (after ref. 4). (c) Regenerative growth curve for quartz aliquots for BG4578 which shows that the Natural SAR ratio is at dose saturation, at about 200-250 Gy, and does not yield a finite age estimate. (d) Regenerative growth curve for quartz aliquots for BG4575 that depicts near dose saturation, at about 200-250 Gy, and does not yield a finite age estimate.

**References :**

1. Murray, A. S. & Wintle, A. G. The single aliquot regenerative dose protocol: potential for improvements in reliability. *Rad. Measur.* **37**, 377-381 (2003).

2. Wintle, A.G., & Murray, A.S. A review of quartz optically stimulated luminescence characteristics and their relevance in single-aliquot regeneration dating protocols. *Rad. Measur.* **41**, 369-391 (2006).

3. Mejdahl, V. & Christiansen, H.H. Procedures used for luminescence dating of sediments. *Boreas* **13**, 403-406 (1994).

4. Galbraith, R. F. & Roberts, R. G. Statistical aspects of equivalent dose and error calculation and display in OSL dating: An overview and some recommendations. *Quat. Geochron.* **11**, 1-27 (2012).

5. Durcan, J.A., & Duller, G.A.T. The fast ratio: A rapid measure for testing the dominance of the fast component in the initial OSL signal from quartz. *Rad. Measur.* **46**, 1065-1072 (2011).

6. Galbraith, R.F., Green, P.F. Estimating the Component Ages in a Finite Mixture. *Nucl. Tracks Rad. Meas.* **17**, 197-206 (1990).

7. Galbraith, R.F., Roberts, R.G., Laslett, G.M., Yoshida, H. & Olley, J.M. Optical dating of single and multiple grains of quartz from Jinmium rock shelter, northern Australia, part 1, Experimental design and statistical models. *Archaeometry* **41**, 339-364 (1999).

8. Wright, D., Forman, S. L., Waters, M. & Ravesloot, J. Holocene eolian activity as a proxy for broad-scale landscape change on the Gila River Indian Community, AZ. *Quat. Res.* **76** (1), 10-21 (2011).

9. Peng, L, & Forman, S. L. LDAC: An Excel-based program for luminescence equivalent dose and burial age calculations. *Ancient TL* 37 (2), 21-40 (2019).

10. Arnold, L. J., & Roberts, R. G. Stochastic modelling of multi-grain equivalent dose (De) distributions: Implications for OSL dating of sediment mixtures. *Quat. Geochron.* **4**, 204-230 (2009).

11. Fain, J., Soumana, S., Montret, M., Miallier, D., Pilleyre, T., & Sanzelle, S. Luminescence and ESR dating-Beta-dose attenuation for various grain shapes calculated by a Monte-Carlo method. *Quat. Sci. Rev.* **18**, 231-234 (1999).

12. Guerin, G., Mercier, N. & Adamiec, G. Dose-rate conversion factors: Update. *Ancient TL* **29**(1), 5-8 (2011).

13. Prescott, J.R. & Hutton, J.T. Cosmic ray contributions to dose rates for luminescence and ESR dating: large depths and long-term time variations. *Rad. Measur.* **23**, 497-500 (1994).

14. Yang, X. Scuderi, L.A. Wang, X., Zhang, D., Hongwei L., Forman, S. L., Xu, Q., Wang, R., Huang, W., Yang, S. Groundwater sapping as the cause of irreversible desertification of Hunshandake Sandy Lands, Inner Mongolia, northern China. *Proceedings of the National Academy of Sciences* **112** (3), 702-706 (2015).
